# Autism spectrum disorder and anorexia nervosa: an Italian prospective study

**DOI:** 10.1186/s13052-021-01006-7

**Published:** 2021-03-09

**Authors:** Jacopo Pruccoli, Altea Solari, Letizia Terenzi, Elisabetta Malaspina, Marida Angotti, Veronica Pignataro, Paola Gualandi, Leonardo Sacrato, Duccio Maria Cordelli, Emilio Franzoni, Antonia Parmeggiani

**Affiliations:** 1 Department of Medical and Surgical Sciences, University of Bologna, Bologna, Italy; 2 Regional Centre for Feeding Eating Disorders, Child Neurology and Psychiatry Unit, IRCCS Institute of Neurological Sciences of Bologna, Bologna, Italy

**Keywords:** Autism spectrum disorder, Anorexia nervosa, ADOS-2, AQ, EDI-3, BMI

## Abstract

**Background:**

Potential overlaps exist between psychopathological features of Anorexia Nervosa (AN) and Autism Spectrum Disorder (ASD). The impact of malnutrition on autistic traits in patients with AN should be considered. This study investigates possible associations among the psychopathology of Eating Disorders (EDs), ASD traits and BMI in a group of young patients with AN, using the EDI-3 (Eating Disorder Inventory-3) test and gold-standard measures for ASD.

**Methods:**

Prospective study involving 23 inpatients admitted to an Italian Centre for paediatric ED. ASD traits and ED psychopathology were assessed administering the ADOS-2 (Autism Diagnostic Observation Schedule-2), AQ (Autism Quotient) and EDI-3 tests. Both present and past autistic traits were investigated using different versions of AQ. Correlations were adjusted for BMI, Obsessive Compulsive Disorder (OCD) comorbidity and concurrent antipsychotic treatments.

**Results:**

An ASD diagnosis was possible in 22% of patients. Significant correlations were documented between ASD traits and ED psychopathology: AQ total-Interpersonal problems (IPC) (*p* = 0.041); AQ total-Global psychological maladjustment (GMPC) (*p* = 0.027); AQ social skills-Ineffectiveness (IC) (*p* = 0.018); AQ social skills-IPC (*p* = 0.019); AQ social skills-Affective problems (APC) (*p* = 0.025); AQ social skills-GMPC (*p* = 0.007); AQ attention switching-IPC (*p* = 0.020); ADOS-2 imagination-IC (*p* = 0.035). These correlations were independent of BMI, OCD and antipsychotic treatments.

**Conclusions:**

ASD traits presented high prevalence in a group of young inpatients with AN. These traits were significantly correlated to 4 specific EDI-3 subscales and independent of BMI. This is the first study to investigate the relationship between ASD traits as measured with gold-standard measures, EDI-3 scores, and BMI.

## Highlights

- In young patients with Anorexia Nervosa, scores on specific EDI-3 subscales assessing Eating Disorders psychopathology correlated with specific autistic traits, as measured by the ADOS-2 and AQ.
- EDI-3 Interpersonal Problems (IPC), Affective Problems (APC), Ineffectiveness (IC), and Global Psychological Maladjustment (GPMC) displayed significant correlations with scores for autistic traits, mainly AQ Total, Social Skills, Attention Switching and ADOS-2 imagination scores.
- The relationship between Eating Disorders psychopathology and autistic traits was independent of BMI, OCD comorbidity and antipsychotic treatments.

## Main text

### Introduction and aims

Eating Disorders (EDs) are pathologic conditions characterized by a persistent disturbance of eating or eating-related behaviour, resulting in altered consumption of food and significantly altered psychosocial functioning [1]. Anorexia nervosa (AN) is a severe ED, characterized by significantly low body weight, intense fear of gaining weight, and undue influence of weight and shape on self-evaluation. AN has one of the highest prevalence among EDs: 1.4% for women and 0.2% for men [5]. The incidence of AN requiring inpatient treatment for the age group 10–19 years is, in Italy, 22.8 per 100.000 women and 2.0 per 100.000 men [7]. The lifetime prevalence of psychiatric comorbidities is very high among individuals affected by AN, from 45 to 97% [8]. Among comorbid psychiatric conditions, Autism Spectrum Disorder (ASD) or autistic traits are frequently documented in patients with AN [10].

ASD is a pervasive neurodevelopmental disorder starting in infancy and characterized by difficulties with reciprocal social interaction and by repetitive, restricted behaviours and interests [1]. Ledford and Gast described prevalence rates of problematic feeding behaviours in ASD ranging from 46 to 89% [14]. Food selectivity represents the most predominant feeding problem, affecting approximately 70% of ASD patients. Feeding disorders as selective or scarce feeding may represent early warning signs of ASD and are frequently associated with delay or stagnation of development [20]. Feeding difficulties should be investigated and considered together with the analysis of anthropometric parameters in children with ASD [21]. In 2013, Huke and colleagues conducted the first systematic review and meta-analysis investigating the prevalence of ASD among patients with AN, and reported an average prevalence of 22.9% [9]. More recent reviews have confirmed these findings [19, 27]. A second meta-analysis [27] reported significantly greater autistic traits in AN patients than in healthy controls, although the latter did not meet the cut-off criteria for a diagnosis of ASD. This study, however, did not report on the impact of BMI values in scores for ASD. All the reviews we consulted seem to indicate a higher prevalence of autistic traits and ASD in AN patients than in the general population.

Many common aspects between the neuropsychological profiles and the emotional and social functioning in AN and ASD make the potential link between the two disorders a subject of interest [28]. Regarding empathy, fantasy and imagination, a review reports very similar profiles between patients with ED and subjects with ASD [11]. Theory of mind, namely the ability to infer the mental states of others, has been shown to present comparable impairment in ASD and AN [15]. On the other hand, different researchers have also stated that malnutrition in patients with AN can determine mental rigidity, obsessive behaviours, and ritualism very similar to ASD symptoms, and that these characteristics may then improve with normal nutrition [17]. Clinicians and researchers should investigate whether autistic traits in AN patients are an epiphenomenon of acute metabolic modifications or rather, specific neurodevelopmental characteristics.

Psychopathological traits are more compromised when EDs overlap with ASD, especially regarding disturbances of thinking, cognitive inflexibility, ritualistic behaviour, mood disorders, phobic aspects, and social impairment. These aspects do not seem to correlate with the severity of malnutrition or BMI [25, 26]. At present there are no protocols to treat patients with an ED associated with ASD or autistic traits: more studies are necessary to clarify this topic and to be able to treat this group of patients with evidence-based guidelines [12].

The aim of the study here described was to investigate the relationship between ASD traits, ED psychopathology and BMI in a population of young inpatients with AN at an Italian Regional Centre for Feeding and Eating Disorders.

## Materials and methods

Twenty-three inpatients were enrolled in the study. Inclusion criteria were: a primary diagnosis of AN according to DSM-5 criteria; an age between 11 and 17 years at the moment of hospitalisation; the presence of one or more symptoms suggestive of ASD, such as: obsessive-compulsive traits, social withdrawal, scarce emotional reciprocity, shared attention deficit, narrow interests, ritualistic and stereotyped behaviour, verbal communication impairment. An exclusion criterion was: being unable to undergo a thorough clinical evaluation including all selected standardised measures. Diagnostic procedures for AN and autistic traits were carried out by clinical psychologists and child neuropsychiatrists from the same Centre where the study was conducted.

A complete clinical evaluation of each participating patient investigated familial and personal history, mental status, anthropometric measures and medical complications of AN. Information was collected regarding the age of onset of AN and symptoms suggestive of ASD, and the natural history of current ED and comorbid conditions.

The Eating Disorder Inventory-3 (EDI-3) [6], completed upon admittance to our Centre, was also collected: this self-assessment questionnaire is routinely employed in the diagnosis of EDs, as it focuses on the psychological domains, which have been proven to be more compromised in ED patients. The result is expressed in the form of six Composite scores: Eating Disorder Risk (EDRC), Ineffectiveness (IC), Interpersonal Problems (IPC), Affective Problems (APC), Overcontrol (OC), Global Psychological Maladjustment (GPMC). These scores are the combination of 12 subscales: Drive for Thinness, Bulimia, Body Dissatisfaction, Low Self-Esteem, Personal Alienation, Interpersonal Insecurity, Interpersonal Alienation, Interoceptive Deficits, Emotional Dysregulation, Perfectionism, Asceticism, Maturity Fears.

Two certified psychologists (V.P. and M.A.) examined each patient for ASD with the Autism Diagnostic Observation Schedule-2 (ADOS-2, Module 4 for adolescents) [16]. The ADOS-2 represents the gold-standard exam for the diagnosis of ASD [18]. Module 4 is used to assess adolescents and adults without language impairment. It expresses a score in the domains of language and communication, reciprocal social interaction, imagination, creativity, stereotyped behaviour, and restricted interests. The scores in the domains of language and communication, and reciprocal social interaction are then used to obtain a final score that can correspond to a diagnosis of “Autism”, “Autistic Spectrum” or “Non-Spectrum”.

On the same day, patients and/or parents were administered the Autism Spectrum Quotient (AQ) [3, 23] under the supervision of a trained, research-reliable psychologist or childhood neuropsychiatrist. The test explores five different domains: social skills, attention shifting, attention to detail, communication, and imagination. This study employed two different versions of the AQ for each patient: one was used to assess present symptoms and behaviour (AQ 12–15 years, which is a parent-report test, or AQ 16+ years, which is a self-report test); the other was used to investigate ASD symptoms and behaviour in childhood (AQ 4–11 years, a parent-report test). This choice enabled us to detect the possible presence of ASD features immediately after their typical onset age and before the appearance of an overlaying AN symptomatology.

### Statistical analysis

All statistical analyses were conducted using JASP, version 0.13.1 for Windows. Adopted alpha error rate was 0.05 (two-tailed), with conservative statistical power of 95%. Descriptive statistics for demographic and clinical variables were calculated for the whole sample, considering means and standard deviations. Descriptive analyses and comparisons for total scores and subscales of the ADOS-2, AQ and EDI-3 were performed. Pearson’s or Spearman’s rho correlations were calculated for selected clinical variables (BMI, EDI-3 scores, AQ-scores, ADOS-2 scores). Shapiro-Wilk and Levene tests were used to assess normality of data distribution and homogeneity of variance. Analyses of covariance (ANCOVA) and logistic regressions controlling for BMI, OCD comorbidity and concurrent treatment with antipsychotics were performed to investigate significant correlations between EDI-3 scores and ASD measures.

### Ethical considerations

The study was approved by the local Ethical Committee (protocol code: ASD-AN-18). Written informed consent was obtained.

## Results

### Demographics

Twenty-three patients were enrolled in the study. Twenty were females (87%). The age range was 13 to 19 years (mean age 15.8 years +/− 1.5). Mean BMI at evaluation was 16.8 +/− 2.1; mean BMI at admittance was 14.2 +/− 1.7. Sixteen patients (70%) were treated with psychiatric drugs. One patient (4%) presented a slight delay in language acquisition. Three patients (13%) had a family history of AN, while none had a family history of ASD. OCD was present in 16 patients (70%), depressive mood in 8 (35%), self-harming in 4 (17%), anxiety in 3 (13%), traits of Bipolar Disorder in 2 (9%).

### ADOS-2

The mean ADOS-2 total score was 4,1 (±5,0). The mean scores for the single subscales were: Communication: 1,3/8 (±1,7); Reciprocal social interaction: 2,2/14 (±3,2); Imagination/Creativity: 0,4/2 (±0,5); Stereotyped behaviour and restricted interests: 0,2/8 (±0,7). Three patients (13%) were classified in the “Autism” category, 2 as “Autistic Spectrum” and 18 as “Non-Spectrum”. Five patients in total (22%) scored, therefore, above-threshold on the ADOS-2. BMI was not significantly correlated with either ADOS-2 total score or any subscale.

### AQ

The Autism Spectrum Quotient (AQ) regarding the present symptoms was administered in both the 16+ years and in the 12–15 years versions, depending on the age of the patient. The mean total score in our sample was 21,9/50 (±7,8). The mean scores for the single subscales were: Social Skills 4/10 (±2,9); Attention Switching: 5,9/10 (±1,9); Attention to Detail: 5,6/10 (±2,3); Communication: 3,6/10 (±2,1); Imagination: 2,9/10 (±1,8). Out of 23, 2 patients (9%) scored clinically significant for ASD, 4 patients (17%) scored borderline clinically significant for ASD, and 17 patients (74%) scored as non-clinically significant for ASD. BMI was not significantly correlated with either AQ-present total score or any subscale.

The AQ 4–11 years test was administered to the parents of the patients in order to collect data on the symptoms and behaviours that occurred in the patients’ childhood. The mean total score in our sample was 54,8/150 (+/− 22,4). The mean scores for the single subscales were: Social Skills: 11,7/30 (+/− 7,0); Attention Switching: 13,1/30 (+/− 5,8); Attention to Detail: 14,3/30 (+/− 4,8); Communication: 8,9/30 (+/− 5,9); Imagination: 8,9/30 (+/− 4,1). Out of 23, 6 patients (26%) scored clinically significant for ASD, 2 (9%) scored borderline clinically significant for ASD, and 15 (65%) scored as non-clinically significant for ASD. BMI was not significantly correlated with either AQ-past total score or any subscale.

Upon comparing present and past versions of the AQ, the mean scores of all patients at single items and subscales show no substantial modification from past to present. With the exception of the “social skills” and “attention to detail” subscales, all past and present versions of subtests show strongly significant correlations (Total: *p* < 0.001; Attention switching: *p* = 0.009; Communication: *p* = 0.004; Imagination: *p* = 0.005).

### ADOS-2 and AQ

Five patients out of 23 (22%) had a diagnosis of “Autism” or “Autism Spectrum” on the ADOS-2. Only one of these patients had corresponding high scores on both AQ questionnaires. Two patients scored above-threshold on the ADOS-2 and AQ past.

### ADOS-2, AQ and EDI-3

The analysis of correlations between EDI-3 scores and the tests for ASD (ADOS-2 and AQ) revealed a series of statistically significant correlations. ADOS-2 total scores correlated with the EDI-3 IC subscale. Significant correlations between ADOS-2 and EDI-3 subscales are shown in Table 1. The AQ-present total score and a series of AQ-present subscales (Social Skills, Attention Switching, Communication) were positively associated with 3 EDI-3 subscales (IPC, APC, GMPC). Correlations are shown in Table 2. AQ-past total scores showed no significant correlation with EDI-3 subscales. Significant correlations between EDI-3 subscales and ASD measures were assessed by performing analyses of covariance (ANCOVA) and logistic regressions controlling for potential confounding factors, namely BMI, OCD comorbidity, and concurrent treatment with antipsychotics. The age at the moment of the evaluation and a concurring treatment with antidepressants, two additional potential confounding factors, resulted not significantly correlated with the scores of the EDI-3, ADOS-2, or AQ tests in our sample.

**Table 1:** EDI-3 domain scores compared with ADOS-2 domain scores (*p*-values)

	EDI-3	EDI-3	EDI-3	EDI-3	EDI-3	EDI-3
	EDRC	IC	IPC	APC	OC	GPMC
ADOS-2 Total	0.164	***0.017***	0.084	0.296	0.420	0.173
ADOS-2 Communication	0.486	0.267	0.297	0.744	0.483	0.925
ADOS-2 Social Interaction	0.356	0.140	0.449	0.424	0.553	0.433
ADOS-2 Imagination and creativity	***0.026***	***0.007***	0.076	0.390	0.056	0.089
ADOS-2 Repetitive behaviours	0.616	0.873	0.334	0.121	0.400	0.192

Statistically significant correlations marked in bold, underlined and italicized

*Abbreviations*: *ADOS-2* Autism Diagnostic Observation Schedule-2nd Edition, *EDI-3* Eating Disorder Inventory-3, *EDRC* Eating Concerns, *IC* Ineffectiveness, *IPC* Interpersonal Problems, *APC* Affective Problems, *OC* Overcontrol, *GPMC* Global Psychological Maladjustment

**Table 2:** EDI-3 domain scores compared with AQ-adolescence domain scores (*p*-values)

	EDI-3	EDI-3	EDI-3	EDI-3	EDI-3	EDI-3
	EDRC	IC	IPC	APC	OC	GPMC
AQ-A Total	0.174	0.075	***0.019***	***0.043***	0.095	***0.023***
AQ-A Social Skills	0.339	***0.038***	***0.022***	***0.007***	***0.019***	***0.003***
AQ-A Attention Switching	0.332	0.059	***0.018***	0.267	0.319	0.105
AQ-A Attention to detail	0.106	0.351	0.413	0.379	0.292	0.300
AQ-A Communication	0.696	0.395	0.089	***0.038***	0.267	0.061
AQ-A Imagination	0.430	0.961	0.381	0.876	0.744	0.955

Statistically significant correlations marked in bold, underlined and italicized

*Abbreviations*: *AQ-A* Autism Quotient, Adolescence version, *EDI-3* Eating Disorder Inventory-3, *EDRC* Eating Concerns, *IC* Ineffectiveness, *IPC* Interpersonal Problems, *APC* Affective Problems, *OC* Overcontrol, *GPMC* Global Psychological Maladjustment

After controlling for BMI, OCD comorbidity and concurrent treatment with antipsychotics, the following correlations resulted significant: AQ total-IPC (F(1,17)=4.894, *p* = 0.041); AQ total-GMPC (F(1,17)=5.854, *p* = 0.027); AQ social skills-IC (F(1,17)=6.924, *p* = 0.018); AQ social skills-IPC (F(1,17)=6.675, *p* = 0.019); AQ social skills-APC (F(1, 17)=6.086, *p* = 0.025); AQ social skills-GMPC (F(1,17)=9.545, *p* = 0.007); AQ attention switching-IPC (F(1,17)=6.574, *p* = 0.020); ADOS-2 imagination-IC (OR = 1.062, 95% IC = 0.004–0.116, *p* = 0.035). Adjusted correlation between ADOS-2 imagination and EDRC was marginally non-significant (OR = 1.960, 95% IC = 0.000–0.125, *p* = 0.050). The following correlations, after adjusting for BMI, OCD comorbidity and concurrent treatment with antipsychotics, resulted non-significant: ADOS-2 total-IC, AQ total-APC, AQ communication-APC.

## Discussion

This is the first study comparing ASD traits, which were evaluated with gold-standard measures (ADOS-2 and AQ tests), to EDI-3 scores and BMI in a sample of young patients hospitalized for AN. The investigation of ASD traits in childhood with the employment of the AQ “past version” represents a second new addition to the literature on the subject. Along with the quantitative measurement, a multidimensional clinical evaluation of the patient was always taken into account since our Centre specializes in the diagnosis and treatment of both ASD and AN.

Five patients, out of the 23 assessed (22%), obtained scores on the ADOS-2 compatible with a diagnosis of ASD. On the whole, 12 patients out of 23 (52%) scored above clinical thresholds for ASD in at least one test (ADOS-2, AQ childhood and adolescence). Notably, the patients, whom we analysed for this study, were selected because they exhibited autistic features. The rationale for this choice was to explore and confirm possible criteria to select AN patients to be screened for ASD in clinical practice.

Our findings on the prevalence of ASD symptoms investigated with the ADOS-2 in patients with EDs are consistent with most of previously published research. In particular, a recent systematic review found a 26.5% prevalence of ASD in patients with EDs [19]. A meta-analysis addressing the prevalence of ASD symptomatology among patients with AN found a wide spectrum of results, ranging from 4 to 52% [28].

Our finding of 26% patients scoring above threshold for ASD in the AQ adolescence version is also consistent with previous research. Specifically, the mean score obtained in our population (21.9) confirms the results reported by a previous systematic review [27]. We documented strong correlations between the two versions of the AQ test, that is, total scores, as well as 4 out of 6 pairs of subscales, showed significantly correlated results between the childhood and adolescence version of the test. This could indicate that ASD traits pre-existed the onset of the ED. In this regard, ADOS-2 and AQ-adolescence total scores and subtests showed no direct correlation with the patients’ BMI. These findings suggest that autistic traits documented in adolescent patients with AN may not be due solely to the severity of their condition during clinical evaluation, which confirms and expands on existing evidence [24].

Correlation measures between tests for ASD and indicators of EDs showed that scores obtained at specific EDI-3 subscales, particularly IPC, APC and GMPC, were significantly correlated with AQ and ADOS-2 scores: this could suggest an association between ED psychopathology as assessed with EDI-3 and ASD traits. Analysis of covariance (ANCOVA) and logistic regressions adjusted for BMI, OCD comorbidity and concurrent antipsychotic treatment confirmed the majority of these findings. These results corroborate and broaden previous data [4] for they show correlations between subthreshold ASD symptoms and selected EDI-2 subscales. In particular, the authors found Interpersonal Distrust EDI-2 subtest to be significantly correlated with total scores obtained on the Adult Autism Subthreshold Spectrum, and in the Non-verbal communication and Inflexibility and adherence to routine domains. However, the ADOS-2 schedule was not adopted in this study. More generally, these results corroborate several previous studies, showing difficulties in social interaction and low levels of social skills in patients with AN [2, 13, 22].

This study has some limitations. Only 23 cases were analysed; on this basis, no far-reaching conclusion can be drawn. This is a relatively small research sample with respect to the larger population that is normally evaluated in a Centre specialized in ED. This is due to our inclusion criteria since, in this preliminary study, we selected to analyse, with a set of standardized measures, a group sharing clinical evidence of ASD traits. The sample of patients that the study considered is non-homogeneous with regard to gender, BMI, psychiatric drugs and interval of time elapsed from discharge to evaluation. The self-report and parent-report questionnaires employed in this study allowed us to include various perspectives in the evaluation of the patients; upon clinical observation, however, some biased attitudes towards the tests were noted, coming from both patients and parents. Nonetheless, most patients and parents were observed to be objective and compliant, and the clinical perspective given by the ADOS-2 permitted a well-rounded investigation.

## Conclusion

While consistent with previous studies, our results are unprecedented in literature and should be investigated in larger samples, given their likely importance for both clinical practice and research. They indicate the possibility that the EDI-3 -- specifically its subscales IPC, APC, and GMPC -- be employed as a preliminary measure to identify and examine patients with AN for ASD traits with gold-standard measures.

## Data Availability

The datasets used and analysed during the current study are available from the corresponding author on reasonable request.

## Abbreviations

ADOS-2: Autism Diagnostic Observation Schedule-2nd Edition
AN: Anorexia Nervosa
AQ: Autism Quotient
ASD: Autism Spectrum Disorder
BMI: Body Mass Index
DSM 5: Diagnostic and Statistical Manual of Mental Disorders, Fifth version
EDI-3: Eating Disorder Inventory-3
EDRC: Eating Concerns
IC: Ineffectiveness
IPC: Interpersonal Problems
APC: Affective Problems
OC: Overcontrol
GPMC: Global Psychological Maladjustment

## References

[1] American Psychiatric Association. Diagnostic and statistical manual of mental disorders. 5th ed. Arlington: American Psychiatric Association, 2013.

[2] Anckarsäter H, Hofvander B, Billstedt E, Gillberg IC, Gillberg C, Wentz E (2012). The sociocommunicative deficit subgroup in anorexia nervosa: autism spectrum disorders and neurocognition in a community-based, longitudinal study. Psychol Med.

[3] Baron-Cohen S, Wheelwright S, Skinner R, Martin J, Clubley E (2001). No title found. J Autism Dev Disord.

[4] Dell’Osso L, Carpita B, Gesi C, Cremone IM, Corsi M, Massimetti E (2018). Subthreshold autism spectrum disorder in patients with eating disorders. Compr Psychiatry.

[5] Galmiche M, Déchelotte P, Lambert G, Tavolacci MP (2019). Prevalence of eating disorders over the 2000–2018 period: a systematic literature review. Am J Clin Nutr.

[6] Garner DM (2004). The eating disorder inventory-3: professional manual.

[7] Gigantesco A, Masocco M, Picardi A, Lega I, Conti S, Vichi M (2010). Hospitalization for anorexia nervosa in Italy. Riv Psichiatr.

[8] Herpertz-Dahlmann B (2015). Adolescent eating disorders. Child Adolesc Psychiatr Clin N Am.

[9] Huke V, Turk J, Saeidi S, Kent A, Morgan JF (2013). Autism spectrum disorders in eating disorder populations: a systematic review. Eur Eat Disord Rev.

[10] Jagielska G, Kacperska I (2017). Outcome, comorbidity and prognosis in anorexia nervosa. Psychiatr Pol.

[11] Kerr-Gaffney J, Harrison A, Tchanturia K (2019). Cognitive and affective empathy in eating disorders: a systematic review and meta-analysis. Front Psychiatry.

[12] Kinnaird E, Norton C, Stewart C, Tchanturia K (2019). Same behaviours, different reasons: what do patients with co-occurring anorexia and autism want from treatment?. Int Rev Psychiatry.

[13] Klump KL, Bulik CM, Pollice C, Halmi KA, Fichter MM, Berrettini WH (2000). Temperament and character in women with anorexia nervosa. J Nerv Ment Dis.

[14] Ledford JR, Gast DL (2006). Feeding problems in children with autism spectrum disorders: a review. Focus Autism Other Dev Disabl.

[15] Leppanen J, Sedgewick F, Treasure J, Tchanturia K (2018). Differences in the theory of mind profiles of patients with anorexia nervosa and individuals on the autism spectrum: a meta-analytic review. Neurosci Biobehav Rev.

[16] Lord C, Rutter M, Dilavore PC, Risi S, Gotham K, Bishop SL (2012). Autism diagnostic observation schedule, second edition (ADOS-2) manual (part 1) modules 1–4.

[17] Mandy W, Tchanturia K (2015). Do women with eating disorders who have social and flexibility difficulties really have autism? A case series. Mol Autism.

[18] National Institute for Health and Clinical Excellence (2011). Autism: recognition, referral and diagnosis of children and young people on the autism spectrum (NICE guideline).

[19] Nickel K, Maier S, Endres D, Joos A, Maier V, Tebartz van Elst L (2019). Systematic review: overlap between eating, autism spectrum, and attention-deficit/hyperactivity disorder. Front Psychiatry.

[20] Parmeggiani A, Corinaldesi A, Posar A (2019). Early features of autism spectrum disorder: a cross-sectional study. Ital J Pediatr.

[21] Parmeggiani A, Pruccoli J. Eating disorders in infants and toddlers. Hidden and lesser-known disordered eating behaviors in medical and psychiatric conditions. Springer; 2021. In press.

[22] Postorino V, Scahill L, De Peppo L, Fatta LM, Zanna V, Castiglioni MC (2017). Investigation of autism spectrum disorder and autistic traits in an adolescent sample with anorexia nervosa. J Autism Dev Disord.

[23] Ruta L, Mazzone D, Mazzone L, Wheelwright S, Baron-Cohen S (2012). The autism-spectrum quotient—Italian version: a cross-cultural confirmation of the broader autism phenotype. J Autism Dev Disord.

[24] Sedgewick F, Kerr-Gaffney J, Leppanen J, Tchanturia K (2019). Anorexia nervosa, autism, and the ADOS: how appropriate is the new algorithm in identifying cases?. Front Psychiatry.

[25] Stewart CS, McEwen FS, Konstantellou A, Eisler I, Simic M (2017). Impact of ASD traits on treatment outcomes of eating disorders in girls: the impact of ASD traits on treatment outcomes of eating disorders in young people. Eur Eat Disord Rev.

[26] Tchanturia K, Adamson J, Leppanen J, Westwood H (2019). Characteristics of autism spectrum disorder in anorexia nervosa: a naturalistic study in an inpatient treatment programme. Autism.

[27] Westwood H, Eisler I, Mandy W, Leppanen J, Treasure J, Tchanturia K (2016). Using the autism-spectrum quotient to measure autistic traits in anorexia nervosa: a systematic review and meta-analysis. J Autism Dev Disord.

[28] Westwood H, Tchanturia K (2017). Autism spectrum disorder in anorexia nervosa: an updated literature review. Curr Psychiatry Rep.

